# Antioxidant activity of polyphenolic compounds isolated from ethyl-acetate fraction of *Acacia hydaspica* R. Parker

**DOI:** 10.1186/s13065-018-0373-x

**Published:** 2018-01-25

**Authors:** Tayyaba Afsar, Suhail Razak, Maria Shabbir, Muhammad Rashid Khan

**Affiliations:** 1 Department of Biochemistry, Faculty of Biological Sciences, Quaid-i-Azam University, Islamabad, Pakistan; 2 Department of Animal Sciences, Faculty of Biological Sciences, Quaid-i-Azam University, Islamabad, Pakistan; 3 Department of Community Health Sciences, College of Applied Medical Sciences, King Saud University, Riyadh, Saudi Arabia; 4 Atta-ur-Rahman School of Applied Biosciences, NUST, Islamabad, Pakistan

**Keywords:** *Acacia hydaspica*, Chromatographic techniques, Catechin isomers, Antioxidant potential

## Abstract

**Background:**

*Acacia hydaspica* belongs to family leguminosae possess antioxidant, anti-inflammatory and anticancer activities. During our search for antioxidant compounds from *A. hydaspica*, we carried out bioassay guided fractionation and obtained antioxidant compounds with free radical scavenging activity.

**Materials and methods:**

The polyphenol compounds in the plant extract of *A. hydaspica* were isolated by combination of different chromatographic techniques involving vacuum liquid chromatography and medium pressure liquid chromatography. The structural heterogeneity of isolated compounds was characterized by high pressure liquid chromatography, MS–ESI and NMR spectroscopic analyses. The antioxidant potential of isolated compounds has been investigated by 1,1-diphenyl-2-picrylhydrazyl (DPPH), nitric oxide scavenging potential, hydroxyl radical scavenging potential, ferric reducing/antioxidant power (FRAP) model systems and total antioxidant capacity measurement.

**Results:**

The isolated compounds show the predominance of signals representative of 7-*O*-galloyl catechins, catechins and methyl gallate. Flash chromatographic separation gives 750 mg of 7-*O* galloyl catechin, 400 mg of catechin and 150 mg of methyl gallate from 4 g loaded fraction on ISCO. Results revealed that **C1** was the most potent compound against DPPH (EC_50_ 1.60 ± 0.035 µM), nitric oxide radical (EC_50_ 6 ± 0.346 µM), showed highest antioxidant index (1.710 ± 0.04) and FRAP [649.5 ± 1.5 µM Fe(II)/g] potency at 12.5 µM dose compared to **C2**, **C3** and standard reference, whereas **C3** showed lower EC_50_ values (4.33 ± 0.618 µM) in OH radical scavenging assay.

**Conclusion:**

Present research reports for the first time the antioxidant activity of polyphenolic compounds of *A. hydaspica*. Result showed good resolution and separation from other constituents of extract and method was found to be simple and precise. The isolation of catechin from this new species could provide a varied opportunity to obtain large quantities of catechin and catechin isomers beside from green tea. Free radical scavenging properties of isolated catechin isomers from *A. hydaspica* merit further investigations for consumption of this plant in oxidative stress related disorders.

**Electronic supplementary material:**

The online version of this article (10.1186/s13065-018-0373-x) contains supplementary material, which is available to authorized users.

## Background

Natural products from medicinal plants, either as pure compounds or as standardized extracts, provide unlimited opportunities for new drug leads because of the unmatched availability of chemical diversity. Due to chemical diversity in screening programs, interest has now grown throughout the world for making therapeutic drugs from natural products [1]. However, the isolation of compounds remains a challenging and a mammoth task. Conventionally, the isolation of bioactive compounds is preceded by the determination of the presence of such compounds within plant extracts through a number of bioassays [2]. The phytochemicals have been found to act as antioxidants by scavenging free radicals, and many have therapeutic potential for the remedy of diseases resulting from oxidative stress [3]. Within the antioxidant compounds, considerable attention has been devoted to plant derived flavonoids and phenolic. Due to the presence of the conjugated ring structures and hydroxyl groups, many phenolic compounds have the potential to function as antioxidants by scavenging or stabilizing free radicals involved in oxidative processes through hydrogenation or complexing with oxidizing species [3]. Moreover, naturally occurring agents with high effectiveness and fewer side effects are desirable as substitutes for chemical therapeutics which have various and severe adverse effects [4]. Plants comprising phenolic constituents, such as phenolic diterpenes, flavonoids, phenolic acids, tannins and coumarins are possible sources of natural antioxidants. Numerous studies have revealed that these natural antioxidants possess numerous pharmacological activities, including neuroprotective, anticancer, and anti-inflammatory activities, and that these activities may be related to properties of antioxidant compounds to prevent diseases by scavenging free radicals and delaying or preventing oxidation of biological molecules [5].

There are different methods to evaluate the in vitro antioxidant capacity of isolated compounds, mixtures of compounds, biological fluids and tissues which involve different mechanisms of determination of antioxidant activity, for example: chemical methods based on scavenging of ROS or RNS, such as nitric oxide (NO∙) radical, DPPH radical and the hydroxyl radical (OH∙) radical [5, 6]. Other assays to determine the total antioxidant power include techniques such as phosphomolybdenum assay (TAC) [6], the ferric reducing/antioxidant power method [7]. Various reaction mechanisms are usually involved in measuring the antioxidant capacity of a complex samples and there is no single broad-spectrum system which can give an inclusive, precise and quantitative prediction of antioxidant efficacy and antiradical efficiency [6], hence, more than one technique is suggested to evaluate the antioxidant capacities [8].

*Acacia* is a diverse genus comprising range of bioactive constituent such as phenolic acids [9], alkaloids [10], terpenes [11], tannins [12] and flavonoids [13], which are responsible for various biological and pharmacological properties like hypoglycaemic, anti-inflammatory, antibacterial, antiplatelet, antihypertensive, analgesic, anticancer, and anti-atherosclerotic due to their strong antioxidant and free radical scavenging activities [14].

*Acacia hydaspica* R. Parker belongs to family “Fabaceae (Leguminosae)”. This species is reported to be common in Iran, India and Pakistan, commonly used as fodder, fuel and wood [15]. The bark and seeds are the source of tannins. The plant is locally used as antiseptic. The traditional healers use various parts of the plant for the treatment of diarrhea; the leaves and the bark are useful in arresting secretion or bleeding. *Acacia hydaspica* possesses antioxidant, anticancer, anti-hemolytic, anti-inflammatory, antipyretic, analgesic and antidepressant potentials [16–18]. Anticancer activity of *A. hydaspica* polyphenols has been determined against breast and prostate cancer [19].

In present study we determined the antioxidant activity of purified compounds from *A. hydaspica* by using five in vitro methods based on different mechanisms of determination of the antioxidant capacity in comparison with reference compounds. The inter-relationships between these methods were also examined for all the tested compounds to check the linearity of activity against different oxidants. Compounds showed linear activity in different antioxidant assays.

## Materials and methods

### Experimental

#### Plant collection

The aerial parts (bark, twigs, and leaves) of *A. hydaspica* were collected from Kirpa charah area Islamabad, Pakistan. Plant specimen was identified by Dr. Sumaira Sahreen (Curator at Herbarium of Pakistan, Museum of Natural History, Islamabad). A voucher specimen with Accession No. 0642531 was deposited at the Herbarium of Pakistan, Museum of Natural History, Islamabad for future reference.

#### Preparation and extraction of plant material

**Fig. 1:**
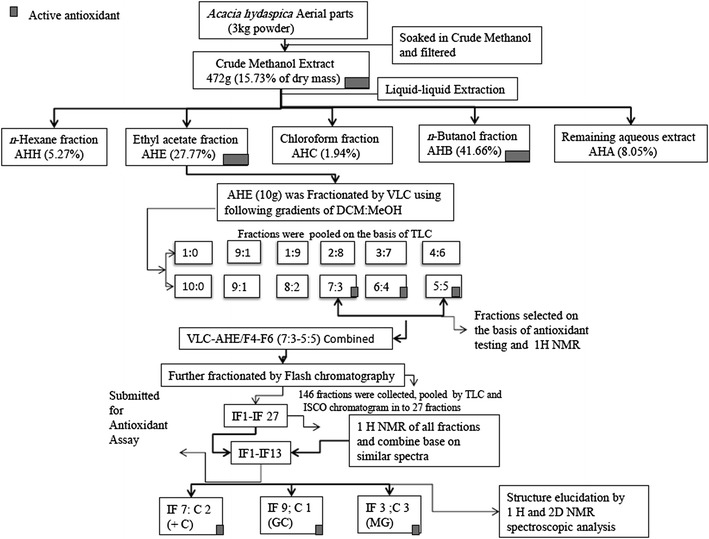
Schematic representation of extraction and isolation of antioxidant compounds from *A. hydaspica* ethyl acetate extract

Partial purification or separation of crude methanol extract was done by solvent–solvent extraction. Briefly 12 g of crude methanol extract was suspended in 500 ml distilled water in separator funnel (1000 ml) and successively partitioned with *n*-hexane, ethyl-acetate, chloroform and *n*-butanol. Each extraction process was repeated three times with 500 ml of each solvent same process was repeated to get enough mass of each fraction to use for chromatographic separation. These solvents with varying polarities theoretically partitioned different plant constituents. The filtrate was concentrated using rotary evaporator (Buchi, R114, Switzerland) and weigh to determine the resultant mass. After this initial partitioning we got four soluble extracts beside crude methanol extract and remaining aqueous extract. The ethyl-acetate (AHE) and butanol (AHB) fractions revealed significant antioxidant potential in various in vitro antioxidant enzyme assays. Estimation of total phenolic content (TPC) and total flavonoid content (TFC) indicate that these AHE and AHB possess high TPC (120.3 ± 1.15,129 ± 2.98 mg Gallic acid equivalent/g dry sample) and TFC (89 ± 1.32, 119 ± 1.04 mg rutin equivalent/g dry sample) respectively [18]. These results prompted us to choose these two extracts for further fractionation and purification of active compounds. Here we report only the isolation and fractionation of ethyl-acetate extract. The scheme of fractionation is summarized in Fig. 1.

### General procedure and reagents

Mass spectrometer with both ESI and APCI spectra were obtained using a TSQ Quantum Triple Quadrupole (Thermo Scientific) ion sources. TLC was conducted on pre-coated silica gel 6OF_254_ plates (MERCK) spots were visualized by UV detection at 254 and 365 nm and Vanillin-HCL reagent followed by heating Semi-preparative HPLC was carried out using a agilent 1260 affinity LC system UV array detection system using a semi-preparative column (Vision HT^™^ classic; 10 μm, 250 × 10 mm). Flash liquid chromatography was carried on Combi-flash Teledyn ISCO (using Redisep column 40 g silica, mobile phase was dichloromethane:methanol (DCM:MeOH), flow rate 15 ml/min) with an ISCO fraction collector. Silica gel (230–400 mesh; Davisil, W. R. Grace) was used for open-column chromatography or vacuum liquid chromatography (VLC). All pure chemicals were purchased from sigma chemicals. All organic solvents were of HPLC grade. Water was purified by a Milli-Q plus system from Millipore (Milford, MA).

#### Vacuum liquid chromatography

The ethyl-acetate acetate extract (AHE) was fractionated with DCM:MeOH of increasing gradient polarity starting with 100% DCM (dichloromethane) to 100% MeOH (methanol) using vacuum liquid chromatographic (VLC) separation. Briefly 10 g of ethyl-acetate extract was dissolve in DCM, mixed with neutral acid wash (super cell NF) and dried down completely with rotavap. Pack 3/4 volume of glass column used for VLC with silica gel and load dried extract sample over the silica layer. After VLC separation, ethyl acetate extract sample was fractionated into 12 fractions of DCM:MeOH in the following gradients; 1:0, 9:1, 8:2, 7:3, 6:4, 5:5, 4.5:5.5, 4:6, 3.5:6.5, 3:7, 2:8, 1:9, 0:1 (v/v). The 7:3 to 5:5 (DCM:MeOH) eluents (VLC-AHE/F3–F4) were mingled according to their TLC and ^1^H-NMR spectra similarity subjected to flash chromatography for further purification of the target compounds.

#### Flash liquid chromatography

VLC-AHE/F4–F6 (4 g/mixed in acid wash/dried) was loaded on Combi-flash Teledyn ISCO. Specifications of run are as follow.

Redisep column: 40 g silica, flow rate: 15 ml/ml, solvent A: dichloromethane (DCM), solvent B: methanol (MeOH), wavelength 1 (red): 205 nm, wavelength 2 (purple): 254 nm all wavelength (orange 200–780 nm) was monitored at all wavelengths (200–780 nm) with Peak width 2 min, and Thresh hold 0.02 AU. Air purge was set at 1 min peak tube volume: 5 ml, nonpeak tube volume 15 ml and loading type solid. 146 fractions collected with ISCO were pooled into 27 fractions according to their TLC and ISCO chromatogram spectral peaks. ^1^HNMR fraction indicated the presence of three pure compounds (**C1**, **C2** and **C3**).

#### High performance liquid chromatography

Chromatographic analysis was carried out to check the purity of isolated compounds by using HPLC–DAD (Agilent USA) attached with Grace Vision Ht C18 column (Agilent USA) analytical column. Compounds stock solutions were prepared in methanol, at a concentration of 0.5 mg/ml. Samples were filtered through 0.45 μm membrane filter. Briefly, mobile phase A was H_2_O (prepared by a Milli-Q water purification system (Millipore, MA, USA) and mobile phase B was acetonitrile. A gradient of time was set as; 0–5 min (isocratic run) for 85% A in 15% B, 5–25 min for 15–100% B, and then isocratic 100% B till 30 min was used. The flow rate was 1 ml/min and injection volume was 20 μl. All the samples were analyzed at 220, 254, 280, 330, and 360 nm wavelengths. Every time column was reconditioned for 10 min before the next analysis. All chromatographic operations were carried out at ambient temperature.

#### % content of isolated compounds

The total content of each isolated compound was expressed as a percentage by mass of the sample.

#### Nuclear magnetic resonance spectroscopy (NMR)

^1^H- and ^13^C-NMR spectrum for all compounds was recorded on a CDD NMR instrument: Varian 600 MHz (^1^H and ^13^C frequencies of 599.664 and 150.785 MHz, respectively) at 25 °C using triple resonance HCN probe: for 1-D proton spectra and proton-detected experiments such as COSY, NOESY, and HMQC. Probe signal-to-noise specifications: ^1^H 1257:1 and broadband switchable probe was used for ^13^C. Chemical shifts were given in *δ* value Spectra of all compounds were obtained in methanol-d4 and DMSO-d6, typically 3–10 mg in 0.4 ml. Conventional 1D and 2D Fourier transform techniques were employed as necessary to achieve unequivocal signal assignments and structure proof for all compounds independently. In addition to 2D shift-correlation experiments (H–H COSY with long-range connectivity’s; C–H correlation via 1^*J*^CH), extensive use was made of ^1^H-coupled ^13^C spectra and selective ^1^H-decoupling to determine long range ^*J*^CH coupling constants and to assign all quaternary carbons unambiguously (DEPTH). Where necessary, stereo-chemical assignments were made with 2D ROESY and NOESY experiments. Detailed analysis of resolution enhanced spectra (Peak picking, integration, multiplet analysis) was performed using ACD/NMR processor (Advanced Chemistry Development, Inc). ^1^H and ^13^C chemical shifts are reported in ppm relative to DMSO-d6 (*δ* 2.5 and *δ* 39.5 for ^1^H and ^13^C respectively), CD3OD (*δ* 3.31, 4.78 for ^1^H and *δ* 49.2 for ^13^C) or internal standard Me_4_Si (TMS, *δ* = 0.0). The NMR spectra and chemical shifts of isolated compounds are matched with published data.

#### Antioxidant capacity determination assays

An amount of 10 mM stock solution of each compound and positive controls [Ascorbic acid, butylated hydroxytoluene (BHT) and Gallic acid] were prepared in 1 ml of solvent according to the assay protocol. These solutions were further diluted to get (0–100 µM) concentration. Positive control varied according to assay requirement.

### Radical scavenging activity

#### DPPH radical scavenging activity assay

The DPPH assay was done according to the method previously describe with slight modifications [20]. The stock solution was prepared by dissolving 24 mg DPPH with 100 ml methanol (80%) and then stored at 20 °C until needed. The working solution was obtained by diluting DPPH solution with methanol to obtain an absorbance of about 0.751 ± 0.02 at 517 nm using the spectrophotometer. An aliquot of 1 ml aliquot of this solution was mixed with 100 μl of the samples at varying concentrations (0–100 µM). The mixture was mixed vigorously and allowed to stand at room temperature in the dark for 10 min. The absorbance of the solution was measured at 517 nm using a UV-1601 spectrophotometer (Shimadzu, Kyoto, Japan). Ascorbic acid was used as a reference compound. The decrease in absorbance was correlated with the radical scavenging potential of test samples. The percentage of inhibition was calculated as follow

$${\text{DPPH scavenging }}\left( \% \right) = \left[ {\frac{{{\text{A}}0 - ({\text{A}}1 - {\text{As}})}}{{{\text{A}}0}}} \right] \times 100.$$

where 0 is the absorbance of the DPPH solution, 1 is the absorbance of the test compound in the presence of DPPH solution, and is the absorbance of the compound solution without DPPH. Each sample was analyzed in triplicate. The EC50 value was calculated by a graphical method as the effective concentration that results in 50% inhibition of radical formation [35].

#### Non site-specific hydroxyl radical scavenging activity

The hydroxyl radical-scavenging activity was monitored using 2-deoxyribose method of Halliwell et al. [21]. Phosphate buffer saline (0.2 M, PH 7.4) was used as a solvent in this assay. Sample solution (0–100 µM) was mixed with assay mixture containing 2.8 mM 2-deoxyribose, 20 mM ferrous ammonium sulphate solution, 100 µM EDTA in a total volume of 1 ml of solvent buffer (0.2 M phosphate buffer saline, PH 7.4). Ferrous ion solution and EDTA were premixed before adding to the assay mixture. The reaction was initiated by the addition of 100 µl of 20 mM H_2_O_2_ and 100 µl of 2 mM Ascorbic acid and incubated at 37 °C for 15 min. Then, thiobarbituric acid solution (1 ml, 1%, w/v) and trichloroacetic acid solution (1 ml, 2%, w/v) were added. The mixture was boiled in water bath for 15 min and cooled in ice, and its absorbance was measured at 532 nm. All experiments involving these samples were triplicated. The scavenging activity were calculated by following formula.

$${\text{Radical} - \text{scavenging capacity }}\left( \% \right) = \left[ {\frac{{{\text{Control absorbance}} - {\text{sample absorbance}}}}{\text{control absorbance}}} \right] \times 100$$

EC50 values, which represent the concentration of sample that caused 50% hydroxyl radical-scavenging activity, were calculated from the plot of inhibition percentage against sample concentration. BHT was used as a positive control.

#### Nitric oxide radical scavenging activity

The interaction of isolated compounds with nitric oxide was accessed by nitrite detection method as previously describe [22]. Nitric oxide was generated with Sodium-nitroprusside previously bubbled with and measured by the Greiss reaction. 0.25 ml of sodium-nitroprusside (10 mM) in phosphate buffer saline was mixed with 0.25 ml of different concentrations (0–100 µM) of compounds and incubated at 30 °C in dark for 3 h. After incubation 0.25 ml of Greiss reagent A (1% sulphanilamide in 5% phosphoric acid) was added and kept at 30 °C for 10 min. After incubation, 0.25 ml of Greiss reagent B (0.1% N 1-naphthylethylenediamine di-hydrochloride) was added mixed and incubated for 20 min. The absorbance of chromophore form during the diazotization of nitrite with sulphanilamide and subsequent coupling with naphthyl-ethylenediamine was read at 546 nm. The same reaction mixture without extract was served as control

$$\% {\text{ inhibition}} = \left[ {1 - \frac{\text{sample absorbance}}{\text{control absorbance }}} \right] \times 100.$$

Rutin was used as a positive control.

### Determination of antioxidant activity

#### Total antioxidant capacity (TAC) (phosphomolybdate assay)

The total antioxidant capacity of compounds was investigated by phosphomolybdate method of Afsar et al. [18]. An aliquot of 100 µl of each sample was mixed with 1 ml of reagent (0.6 M H_2_SO_4_, 0.028 M sodium phosphate, and 0.004 M ammonium molybdate) and incubated for 90 min at 95 °C in a water bath. Absorbance was recorded at 765 nm after the mixture cooled to room temperature.

Ascorbic acid served as positive control.

#### Ferric reducing antioxidant power (FRAP)

A slightly modified method of Benzei and Strain [7] was adopted to estimate the ferric reducing ability of compounds isolated from *A. hydaspica*. Ferric-TPTZ reagent (FRAP) was prepared by mixing 300 mM acetate buffer, pH 3.6, 10 mM TPTZ in 40 mM HCl and 20 mM FeCl_3_·6H_2_O at a ratio of 10:1:1 (v/v/v). Compounds or reference were allowed to react with FRAP reagent in the dark for 30 min. In order to calculate FRAP values (µM Fe(II)/g) for compounds, linear regression equation for standard (FeSO_4_·7H_2_O) was plotted. The standard curve was linear between 100 and 1000 µM FeSO_4_. Results are expressed as μM (Fe(II)/g) dry mass.

### Statistical analysis

All values are mean of triplicates. The Graph Pad Prism was used for One-way ANOVA analysis to assess the difference between various groups and calculation of EC_50_ values. Difference at p < 0.05 were considered significant. In addition, simple regression analysis on Microsoft excel was performed to seek relationship between different tests.

### Chemistry

#### Compound 1: 7-*O*-galloyl-(+)-catechin

Light green shine crystals (H_2_O), C_22_ H _18_ O_10_. MS/ESI(−) m/z 441.0977 [M−H], 1H-NMR (600 MHz, DMSO-d6), δ 7.04 (H-7, s, galloyl), δ 6.17 (H-8, J = 2.2 Hz), δ 6.11 (H-6, *d*, J = 2.2 Hz), δ 4.61 (H-2, *d*, J = 7.6 Hz), δ 3.88–3.93 (H-3, *m*), δ 2.52 (H-4a, *dd*, J = 16.7 Hz, J = 7.9 Hz), δ 2.71 (H-4b, *dd*, J = 16.3 Hz, J = 5.3 Hz). 13C NMR (methanol-d4-150.79 MHz): δ 27.21 (*t*, C-4), δ 66.941 (*d*, C-3), δ 81.975 (*d*, C-2), δ 100.946, δ 104.52 (each *d,* C-6 and C-8), δ 105.957 (s, C-4a), δ 109.179 (*d*, galloyl C-2 and C-6), δ 113.832, δ 114.548 (each *d*, C-2′ and C-5′), δ 119.201 (s, galloyl C-1), δ 130.656 (s, C-1′), δ 138.88 (s, galloyl, C-4), δ 144.973 (s, galloyl, **C3** and C-5), δ 150.343 (s, C-7), δ 155.354, δ 156.070 (each s, C-5 and C-8a), δ165.734 (s, COO–).

#### Compound 2: Catechin

Light yellow amorphous powder, (H_2_O) (C_15_H_14_O_6_). MS/ESI(−) m/z [M−H]. ^1^H-NMR (DMSO-d6, 600 MHz): δ 5.67 (H-8 *d*, J = 2.3 Hz), δ 5.87 (H-6, *d*, J = 1.8 Hz), δ 4.46 (H-2, *d*, J = 7.6 Hz), δ 3.76–3.82 (H-3, m), δ 2.33 (H-4α, *dd*, J = 16.1 Hz, J = 7.9 Hz), δ 2.64 (H-4β, *dd*, J = 16.4 Hz, J = 5.3 Hz), δ 6.7 (H-2′, *d*, J = 1.8 Hz), δ 6.66 (H-5′, *d*, = 8.2 Hz), δ 6.57 (H-6′, *dd*, J = 8.2 Hz, J = 1.8 Hz). ^13^C-NMR (DMSO-d6-150.79 MHz). δ 28.01 (C-4), δ 66.717 (C-3), δ 81.411 (C-2), δ 94.314 (C-8), δ 95.389 (C-6), δ 99.331 (C-4a), δ 114.026 (C-2′), δ 115.10 (C-5′), δ 118.685 (C-6′), δ 130.870 (C-1′), δ 145.206 (C-4′), δ 146.281 (C-3′), δ 156.317 (C-5), δ 156.317 (C-8a), δ 156.317 (C-7).

#### Compound 3: Methyl gallate

White needle crystals. (C_8_H_8_O_5_). MS/ESI(−) m/z 183.0534 [M−H]. ^1^H-NMR (acetone-D6, 600 MHz),: δ3.79 (3H, s, OCH3), δ 7.11 (2H, s, H-2, H-6); ^13^C NMR (acetone-D6, 150.80 MHz) δ 51.0 (OCH3), δ 108.90 (C-2, C-6), δ 120.91 (C-1), δ 137.76 (C-4), δ 145.12 (C-3, C5), δ 166.27 (C=O).

## Results and discussion

**Fig. 2:**
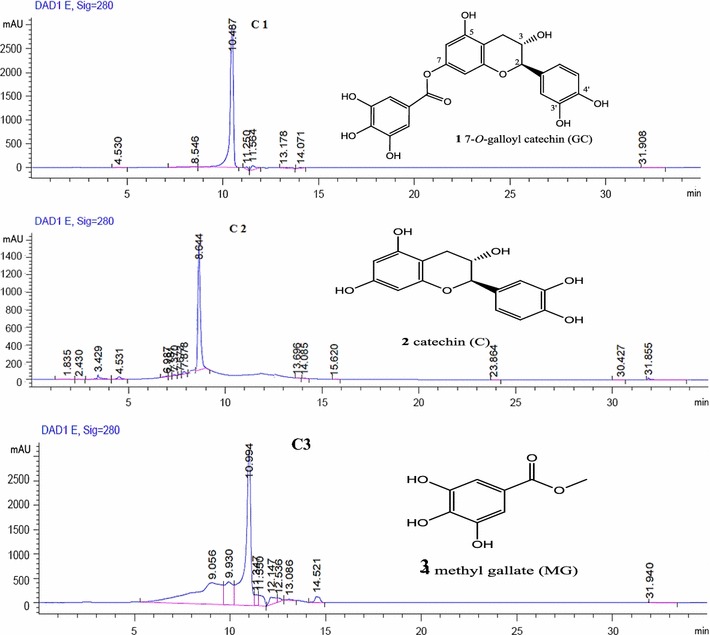
Analytical HPLC chromatogram of **C1**, **C2** and **C3** showing single peaks at 10.487, 8.644 and 10.994 min, and compound structures. Chromatographic conditions: Vision Ht C18 column (5 μm; 10  ×  250 mm, Agilent USA). Mobile phase A (Millipore H_2_O) and mobile phase B (acetonitrile) in gradients: 0–5 min; 15% B in A (isocratic run), 5–27 min; 15–100% B (gradient mode), 27–32 min; 15% B in A (for column equilibration). Flow rate; 1 ml/min, injection volume 20 µl. All compounds showed UV maxima at 280 nm (characteristic of polyphenolic compounds). 7-*O*-galloyl catechin (**C1**), catechin (**C2**), and methyl gallate (**C3**)

The ethyl-acetate fraction of *A. hydaspica* whole plant was fractionated by VLC chromatography and flash chromatography using silica to give several fractions and three pure compounds **C1**, **C2** and **C3**. ISCO chromatogram showed the peaks and pattern of collection of isolated compounds (Additional file 1: Figure S1). Isolated compounds were identified as 7-*O* galloyl catechin (**C1**), catechin (**C2**) [23, 24] and methyl gallate (C**3**) [25] by comparison of their 1D and 2D NMR spectral data with the reported data in the literature (Tables 1, 2; Additional file 2: Figure S2). Figure 2 indicated the Purity of the compounds analyzed by analytical HPLC.

**Table 1:** ^1^H-NMR data of polyphenols isolated from *Acacia hydaspica* (Coupling constant J in Hertz)

Proton	7-*O*-galloyl catechin δ in ppm (**C1**)^a^	(+)-catechin δ in ppm (**C2**)^a^	Methyl gallate δ in ppm (**C3**)^b^
H-2	4.61 (*d*, J = 7.0 Hz)	4.46 (*d*, J = 7.6 Hz)	7.11 (s)
H-3	3.88–3.94 (m)	3.79–3.82 (m)	3.79 (s, OCH3)
H-4α b	2.71 (*dd*, J = 16.3 Hz, J = 5.3 Hz) 2.45 (*dd*, J = 16.5, 7.9 Hz)	2.64 (*dd*, J = 16.4, 5.3 Hz) 2.33 (*dd*, J = 16.1, 7.9 Hz)	–
H-6	6.11 (*d*, J = 2.2 Hz)	5.67 (*d*, J = 2.3 Hz)	7.11 (s)
H-8	6.17 (*d*, J = 2.2 Hz	5.87 (*d*, J = 1.8 Hz)	–
H-2′	6.72 (*d*, J = 1.5 Hz)	6.70 (*d*, J = 1.8)	–
H-5′	6.68 (*d*, J = 8.1 Hz)	6.67 (*d*, J = 8.2 Hz)	–
H-6′	6.60 (*dd*, J = 8.1 Hz, J = 1.5 Hz)	6.57 (*dd*, J = 8.2 Hz, J = 1.8 Hz)	–
OH-3	5.01 (d, J = 5.1 Hz)	4.84 (d, J = 4.7 Hz)	–
Galloyl	7.04 (s)	–	–

Coupling constants (Hz) in parenthesis, ^a^ DMSO-d6 ^b^ indicates acetone–d6. Dashes indicate that given proton is absent the molecule

**Table 2:** ^13^C NMR data of polyphenols isolated from *Acacia hydaspica* ethyl-acetate extract

Carbon	7-*O*-galloyl-catechins δ in ppm (GC; **C1**)^a^	(+)-catechins δ in ppm (C; **C2**)^a^	Methyl gallate δ in ppm (MG; **C3**)^b^
C-1	–	–	120.912
C-2	81.975	81.411	108.901
C-3	66.941	66.717	145.121
C-4	27.211	28.012	137.760
C-4a	105.957	99.331	–
C-6	100.946	94.314	108.901
C-8	104.521	95.389	–
C-5	155.354	156.317	145.120
C-7	150.343	156.317	–
C-8a	156.070	156.317	–
C-1′	130.656	130.870	–
C-2′	113.832	114.026	–
C-3′	–	146.281	–
C-4′	–	145.206	–
C-5′	114.548	115.101	–
C-6′	–	118.685	–
C-1 galloyl	119.201	–	–
C-2 galloyl	109.179	–	–
C-3 galloyl	144.973	–	–
C-4 galloyl	138.881	–	–
C-5 galloyl	144.973	–	–
C-6 galloyl	109.179	–	–
COO–	165.734	–	–
C=O	–	–	166.270
Methyl	–	–	51.012

^a^ DMSO-d6 and ^b^ indicates acetone–d6. Dashes indicate that given carbon is not present in the molecule

### Compound 1

The ^1^HNMR spectrum of **C1** was similar to ^1^HNMR of (+)-catechin except for the additional signal at δ 7.04 (2H, s) due to a galloyl group. The location of the galloyl group was initially deduced to be at either C-5′ OH or C-7′ OH, C-4′ OH, C-3′ OH but not 3 of the catechins moiety from the HMBC spectrum in methanol-d4. In order to determine unequivocally the position of the galloyl group the HMBC was re-perform with DMSO and NOESY data indicate that the stereochemistry of isolated compound as 7-*O*-galloyl-(+)-catechin and which was further authenticated by comparison of the physical data with those reported previously [24, 26]. Consequently, the structure of **C1** was concluded to be 7-*O*-galloyl-(+) catechin.

### Compound 2

The ^1^HNMR spectrum and ^13^C-NMR of **C2** was similar to assignment of catechin signals of those reported in previous literature [27, 28]. Consequently, the structure of **C2** was concluded to be (+) catechin.

### Compound 3

The molecular formula was determined from the MS and 13C NMR. 8 Carbons and five protons attached to carbon were observed in the 13C and ^1^HNMR spectra. In order to determine the position and number of hydroxyl groups, the NMR solvent was shifted to DMSO-d6 as hydroxyl were not seen with acetone-d6. ^1^H-NMR (DMSO-d6, 600 MHz) clearly reveal the presence two hydroxyls at δ9.44 and one hydroxyl at δ9.11. Close examination of the ^1^H and ^13^C NMR spectrum showed a symmetrical molecule with two aromatic protons, δ 7.11 (2H, s, H-2, H-6), three hydroxyl, two hydroxyl at δ C 145.12 (C-3, C-5), and one hydroxyl at δ C 137.76 (C-4), a methyl δ3.79 (3H, s, OCH3) and a ester carbonyl δ 166.27 (C=O). It is consistent with-NMR data have been reported from the literature [14, 15]. The structure (**C3**) revealed to be methyl 3, 4, 5-trihydroxybenzoate or methyl gallate.

### Extractable compound yield

*Acacia hydaspica* ethyl-acetate extract (AHE) yields 187.5 mg/g of **C1**, 100 mg/g of **C2** and 37.5 mg/g of **C3**.

### Determination of anti-radical activity

#### DPPH radical scavenging

**Fig. 3:**
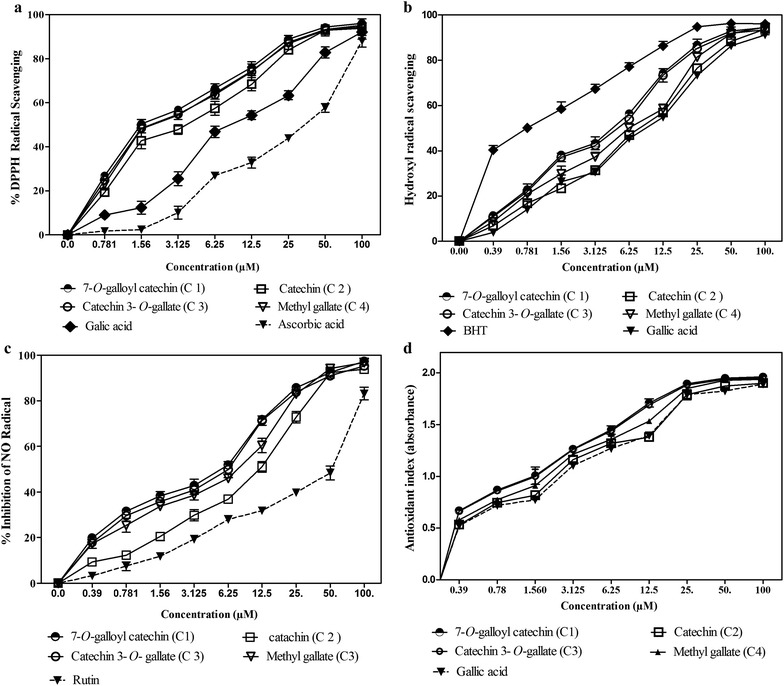
**a** Dose dependent DPPH radical scavenging activity. Ascorbic acid and Gallic acid used as a standard reference. **b** Hydroxyl radical scavenging activity. Butylated hydroxytoluene (BHT) and gallic acid. **c** Dose dependent inhibition of RNS derived from nitric oxide by isolated compounds (**C1**–**C3**) in comparison with standard reference Rutin. **d** Dose dependent increase in total antioxidant capacity (TAC) of isolated compounds. Gallic acid used as standard reference. Values are expressed as mean ± SEM (n = 3). **C1**: 7-*O*-galloyl catechins, **C2**: catechins and **C3**: methyl gallate (**C3**)

The first method, DPPH radical scavenging activity indicates the hydrogen donating ability of compounds. The DPPH free organic nitrogen radical is very stable; contain an odd electron which reacts with compounds that can donate hydrogen atoms. DPPH on accepting electron donated by an antioxidant compound reduces and the purple color is change to yellow. The degree of reduction in absorbance measurement is indicative of scavenging potential of compounds [29]. Thus, we evaluated the free radical-scavenging activity of three polyphenols from *A. hydaspica*. All test compounds exhibited dose dependent quenching of DPPH radical. **C1**, **C2**, and **C3** exhibited the similar antioxidant activities. At a concentration of 100 μM, the scavenging activity of **C1**, **C2** and **C3** reached 96.174 ± 1.95, 93.83 ± 0.85 and 94.527 ± 1.170% respectively, while at the same concentration that of Ascorbic acid and rutin were 87.97 ± 2.654 and 92.160 ± 3.2% respectively. All compounds showed better antioxidant activity than the positive controls (Ascorbic acid and Gallic acid), and the highest DPPH-scavenging activity was shown by compound C**1**, followed by compounds C**3**, and C**2** (Fig. 3a, Table 3). The EC50 value for **C1** was 1.60 ± 0.035 μM which is fivefold more potent than Gallic acid (9.1 ± 0.42 μM) and 22 fold more potent then Ascorbic acid (36.3 ± 0.569 µM). The relative potencies of the compounds were in the order: **C1** > **C3** > **C2** > rutin > Ascorbic acid. Compounds **C2** and **C3** had been investigated on DPPH-scavenging activities previously. The EC_50_ value for catechin (**2**) was 6.24 ± 0.254 µM in the DPPH assay was similar to that reported in Hsu et al. study (EC50 value 6.38) [5, 30]. The EC_50_ value for methyl gallate (**C3**) was 2.92 μM in the DPPH assay, and this data indicate slightly lower EC_50_ value to that reported in Pfundstein’s study (EC_50_ value 4.28 μM) [31]. From these results, it was also possible to make a number of correlations regarding the relationship between the structure of isolated compounds and their DPPH-scavenging activities. Methyl gallate (**C3**) which is the methyl ester of Gallic acid appeared to enhance the bioactivity of Gallic acid (reference compound). It was found that the antioxidant activity of flavan-3-ols isolated from *A. hydaspica* decreased in the following sequence: **C1** > C**2** (i.e., 7-*O*-gallate, 5′-OH > 3-OH, 5′-OH) which is also in good agreement with previously reported data [5]. It appears that as far as the antioxidant activity is concerned, a galloyl group is essential for bioactivity and additional insertion of the hydroxyl group at the 7′ position in the B ring also contributes to the scavenging activities. Comparing the DPPH-scavenging activity of flavan-3-ols (**C1** and **C2**) proven that more phenol groups are central to an intensification of antioxidant activity [5].

**Table 3:** EC_50_ values (concentration causing 50% inhibition) in various antioxidant assays and FRAP potential of *Acacia hydaspica* polyphenols

Compounds	DPPH radical EC50 (µM)	Hydroxyl radical EC50 (µM)	Nitric oxide EC50 (µM)	FRAP µM Fe(II)/g	% (dry weight of AHE extract)
Flavan-3ols					
**C1**	1.60 ± 0.035^a^	4.33 ± 0.618^b^	6 ± 0.346^a^	649.5 ± 1.511^a^	18.75
**C2**	6.24 ± 0.254^b^	8.0 ± 0.635^a^	12.3 ± 0.376^b^	432.9 ± 0.94^b^	10.01
Phenol compound					
**C3**	2.9 ± 0.318^a^	6.25 ± 0.577^a^	7.67 ± 0.577^a^	505.5 ± 2.512^c^	3.75
Standard reference					
BHT	–	0.781 ± 0.115^c^	–	–	–
Ascorbic acid	36.3 ± 0.569^d^	–	–	–	–
Rutin	–	–	53 ± 1.155^c^	–	–
Gallic acid	9.1 ± 0.421^c^	9.67 ± 0.577^a,d^	–	49.5 ± 2.211^c^	–

Values are expressed as mean ± SEM (n = 3); means with superscript with different letters ^(a–d)^ in the row are significantly (p < 0.01) different from each other. Data analyzed by using one way ANOVA followed by Tukeys multiple comparison tests

#### Hydroxyl radical-scavenging activity

ROS constitute a major pathological factor causing many serious diseases, including cancer and neurodegenerative disorders [32]. The generally formed ROS are oxygen radicals, such as hydroxyl radicals and superoxide, and non-free radicals, such as hydrogen peroxide and singlet oxygen. The hydroxyl radical is the most reactive and induces severe damage to adjacent biological molecules [33]. The hydroxyl radical scavenging assay is based on ability of antioxidant to inhibit the formation of the hydroxyl radicals, malondialdehyde (MDA) formation and to prevent the degradation of 2-deoxyribose. Result demonstrated that all tested compounds inhibit hydroxyl radical generation in a dose dependent fashion. The respective EC_50_ values for isolated compounds **C1, C2** and **C**3 were 4.33 ± 0.635, 8.00 ± 0.577 and 6.25 ± 0.618 μM respectively, exhibited greater potency to scavenge hydroxyl radical then Gallic acid (EC_50_ 9.67 ± 0.577 µM) (Fig. 3b, Table 3). However none of tested compound showed better scavenging potential than standard BHT (EC_50_ 0.781 ± 0.115). To our knowledge, the abilities of the compounds **C2**, and **C3** to showed similar potency to scavenge hydroxyl radical to reported in previous studies [5]. From our results, it was also possible to make a number of correlations regarding the relationships between the structures of isolated compounds and their hydroxyl radical-scavenging activities. Methyl gallate (**C3**) seemed to augment the bioactivity of Gallic acid (Reference compound). It was found that the antioxidant activities of flavan-3-ols decreased in the following sequence: **C2** > **C1** (i.e., 3-OH, 5′-OH > 7-*O*-gallate, 5′-OH). This suggests that a galloyl group and *O*-dihydroxy (i.e., catechol) is essential, and 5′-OH is not an important group in antioxidant activity. Comparing the hydroxyl radical-scavenging activities of isolated compounds revealed that the bioactivity decreased in the following sequence: **C1** > **C3** > **C2**. The results suggest that carbonyl, *O*-dihydroxy and galloyl group increased the hydroxyl radical scavenging activity.

#### Inhibition of RNS derived from nitric oxide

Nitric oxide a potent oxidizing radical leads to tissue damage in a number of pathological conditions in humans and experimental animals [34]. Herein, isolated compounds from *A. hydaspica* were examined for their ability to protect against NO-dependent oxidation. Thus, the NO radical-scavenging activities of these isolated compounds were investigated by examining the oxidation of sodium nitroprusside. Figure 3c shows that exposure of nitric oxide generated by sodium nitroprusside to oxygen in the presence of the polyphenols isolated from *A. hydaspica* resulted in a significant inhibition of nitrite ion formation in a dose-dependent manner. The relative EC50 values of compound **C1, C2** and C**3** against RNS derived from nitric oxide are summarized in Table 3, which ranged from 6 to 12.3 µM compared to that of rutin (53.00 ± 1.155 µM). The bioactivity decrease in the following order: GG > MG > C > rutin. The addition of polyphenols significantly inhibited nitric oxide formation even at lower concentrations. Compounds at 25 µM dose showed inhibitory activity, ranging from 85.817±, 83.023± to 72.864± % for MG, GC and C respectively compared to rutin at same dose (39.845 ± 1.48%) as positive control. At a concentration of 100 μM, the scavenging activity of GC, C, and MG reached 97.34 ± 0.982% (p < 0.001), 93.825 ± 1.5 (p < 0.001) and 96.823 ± 1.501% (p < 0.01) respectively indicating significant difference from standard reference rutin (83.163 ± 2.79). These results reveal that the presence of hydroxyl and-carbonyl group in the flavonoid skeleton resulted in high nitric oxide inhibition of compounds. From these results, it was also possible to make a number of correlations regarding the relationship between the structures of isolated compounds and their NO radical-scavenging activities. Methyl gallate (**C3**) appeared to have enhanced the bioactivity then Gallic acid. It appeared that as far as the antioxidant activity was concerned, a galloyl group was essential, while **C3** showed greater bioactivity. It was found that the antioxidant activities of flavan-3-ols decreased in the following sequence: **C1** > **C2** (i.e., 7-*O*-gallate, 5′-OH > 3-OH, 5′-OH). It is well known that nitric oxide has an important role in various inflammatory processes. Sustained levels of production of this radical are directly toxic to tissues and contribute to the vascular collapse associated with septic shock, whereas chronic expression of nitric oxide radical is associated with various carcinomas and inflammatory conditions including juvenile diabetes, multiple sclerosis, arthritis, and ulcerative colitis [35]. The present study showed that GC, C and MG have good nitric oxide scavenging activity then rutin and gallic acid.

#### Total antioxidant capacity (TAC)

Phosphomolybdenum assay principal follows the chemistry of conversion of Mo (VI) to Mo (V) by compounds having antioxidant potential and resulting in the formation of green phosphate/Mo (V) having absorption maxima at 695 nm at acidic PH. TAC assay was used to assess the capacity total antioxidant capacity of isolated compounds compared Gallic acid [36]. Isolated compounds showed good antioxidant index. Total antioxidant capacity (TAC) of compounds increase with increasing concentration of compounds. TAC order of *A. hydaspica* compounds TAC values were in following order; **C1** (1.71 ± 0.040 µM) > **C3** (1.54 ± 0.025 µM) > Gallic acid (1.39 ± 0.004) ~ **C2** (1.379 ± 0.021) at 12.5 µM dose (Fig. 3d). To the best of our knowledge literature is scarce about the total antioxidant activity of 7-*O*-galloyl catechin (**C1**) by phosphomolybedate method. **C1** significantly reduce Mo (VI) to Mo (V) and form a green colored complex of Mo (v) that gives absorbance at 695 nm. Antioxidant index of **C2** is shown to be comparable with Gallic acid (p > 0.05), Methyl ester in **C3** might responsible for significant (p < 0.01) enhancement in TAC capacity as compared to standard Gallic acid. From these results, it was also possible to make a number of correlations regarding the relationship between the structures of isolated compounds, their antioxidant activities and antioxidant index. The transfer of electron or hydrogen depends on the structure of compounds. These results reveal that the presence of hydroxyl and-carbonyl group in the flavonoid skeleton resulted in enhancement of total antioxidant capacity and moreover antioxidant index tested polyphenol compounds isolated from *A. hydaspica* correlated with the number of aromatic hydroxyl groups in the antioxidant assays [37]. It was found that the antioxidant index of isolated compounds decreased in the following sequence: **C1** > **C3** > **C2** (i.e., 7-*O*-gallate, 5′-OH > 3-OH, 5′-OH). The present study showed that **C1**, **C2** and **C3** have good TAC comparable to Gallic acid.

#### FRAP assay

FRAP assay, based on the reduction of ferric tripyridyltriazine complex to its ferrous colored form. The antioxidant activities were measured three times to test the reproducibility of the assays. The Frap assay which measures the ability of isolated compounds to reduce TPTZ-Fe(III) complex to TPTZ-Fe(II) was used to assess the total reducing power of antioxidants [37]. when a Fe^3+^-TPTZ complex is reduced by electron donating antioxidants under acidic conditions, change of absorbance of colorless less Fe^3+^ to blue colored Fe^2+^ form was measured at 593 nm [7]. A higher value indicates higher ferric reducing power. The addition of polyphenols significantly reduces ferric ions to ferrous ions. Tested compounds **C1**, **C2** and **C3** at 12.5 µM dose showed FRAP values of 649.50 ± 1.501, 432.90 ± 0.949 and 505.5 ± 2.500 (µM Fe(II)/g) (Table 3). Results showed that 7-*O*-galloyl catechin (**C1**) has more significant (p < 0.001) FRAP values than catechin (**C2**), methyl gallate (**C3**) and standard reference Gallic acid at same dose; indicating significant electron donating capacity of **C1** in comparison to **C2**, **C3** and Gallic acid. **C2** was less potent then Gallic acid, whereas methyl gallate (**C3**) showed bioactivity slightly but non-significantly enhanced than Gallic acid. Methyl gallate (**C3**) showed bioactivity slightly enhanced than Gallic acid. From these results, it was also possible to make a number of correlations regarding the relationship between the structures of isolated compounds and their FRAP activity. These results reveal that the presence of hydroxyl and-carbonyl group in the flavonoid skeleton resulted in high FRAP potential and reducing ability was concerned the number of aromatic hydroxyl and galloyl group. It was found that the antioxidant activities of isolated compounds decreased in the following sequence: **C1** > **C3** > **C2** (i.e., 7-*O*-gallate, 5′-OH > 3-OH, 5′-OH). The present study showed that 7-*O*-galloyl catechin (**C1**), catechin (**C2**) and methyl gallate (**C3**) have good FRAP reducing potential comparable to Gallic acid.

#### Relationship between different antioxidant variables

Correlation analysis was used to explore the relationships amongst different antioxidant variables measured for compounds. On the basis of simple regression testing, correlation coefficients were calculated among antioxidant assays used in study. Significant linear relationship or high correlation was observed between different antioxidant assay i.e., TAC, FRAP, DPPH, OH and NO radical scavenging assay (Table 4). These results could explained that despite different hydrophilic properties of the substratum used in the different methods, compounds showed a linear activity between different antioxidant assays.

**Table 4:** Relation between antioxidant activity measurements of 3 AH polyphenols using different methods to evaluate the antioxidant activities of isolated compounds from *Acacia hydaspica*

	DPPH^·^	NO^·^	OH^·^
DPPH^·^	NA	–	–
NO^·^	y = 1.3736x + 3.7351 R^2^ = 0.9993	NA	–
OH^·^	y = 0.7411x + 3.5321 R^2^ = 0.9301	y = 0.5354x + 1.5528 R^2^ = 0.9165	NA
TAC	y = − 0.0666x + 1.7807 R^2^ = 0.9264, p < 0.01	y = − 0.0481x + 1.9586 R^2^ = 0.9124	y = − 0.0901x + 2.0993 R^2^ = 0.9999
FRAP	y = − 41.969x + 680.02 R^2^ = 0.8271	y = − 30.177x + 790.87 R^2^ = 0.8073	y = − 59.277x + 896.42 R^2^ = 0.9742

NA indicates same assay no correlation done, Y indicates the regression equation, and R^2^ shows the coefficient of correlation between assays mentioned. – Indicate the same value. Regression analysis done by graph pad prism

Although catechin and methyl gallate were evaluated previously for antioxidant potential by various methods [37]. Nevertheless, the present work provides more information about these features, since five different antioxidant methods were used to analyze the antioxidant capacity of these compounds in comparison with standards i.e., Ascorbic acid, gallic acid, BHT and rutin. In *A. hydaspica* ethyl-acetate extract 7-*O*-galloyl catechin appears to be the major antioxidant compound both in term of yield and activity. These results are in good agreement with the previous report of Zhao et al. [38], which showed that galloyl catechins contributes to the main antioxidant capacity of tea.

## Conclusion

Antioxidant screening of active compounds from unexplored species of *Acacia* genus pave the way for the possible development of natural essences to substitute synthetic ones. There for further investigation for the isolation of compounds from other fractions and their pharmacological evaluations are still required. Moreover the isolation of catechin this new species could provide a new opportunity to obtained catechin beside from green tea. *Acacia hydaspica* provide a source of natural, significantly potent antioxidant constituents that might leads to the prevention of ROS mediated diseases by scavenging free radicals or preventing the oxidation of biomolecules.

## Additional files

**Additional file 1: Figure S1.** ISCO chromatogram showing the peaks of fractions. Arrows indicate the pooling of fractions which leads to pure compounds.

**Additional file 2: Figure S2.** 1H-NMR spectrum of *A. hydaspica* compounds.
