# Supplementary figures and images for: Antioxidant activity of polyphenolic compounds isolated from ethyl-acetate fraction of Acacia hydaspica R. Parker
Source: Chem Cent J. 2018 Jan 25;12:5. doi: 10.1186/s13065-018-0373-x (PMC5785459; doi:10.1186/s13065-018-0373-x)

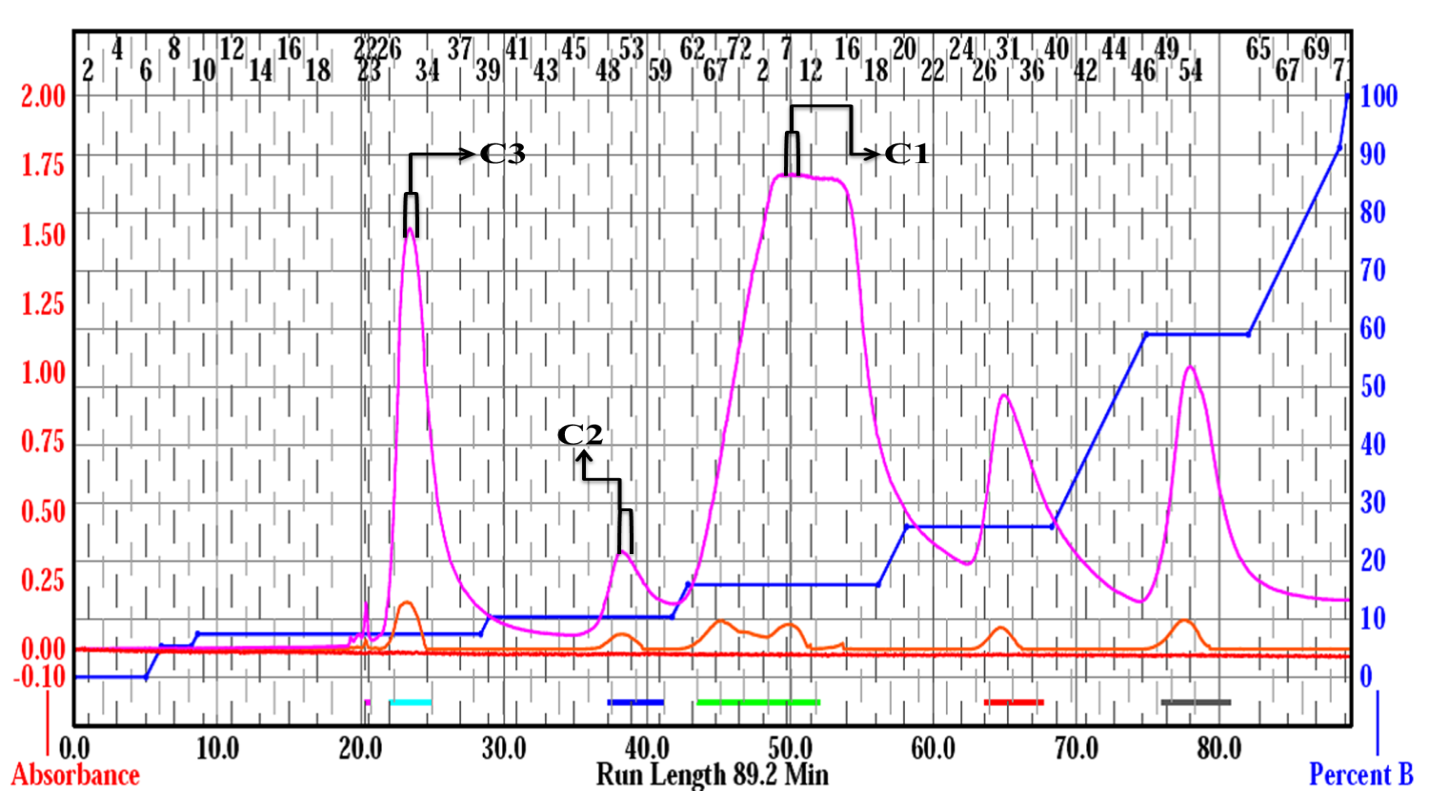

Supplement: Supplementary file 1 — Additional file 1: Figure S1. ISCO chromatogram showing the peaks of fractions. Arrows indicate the pooling of fractions which leads to pure compounds. [file 13065_2018_373_MOESM1_ESM.docx]
