# Supplementary material for: Antioxidant activity of polyphenolic compounds isolated from ethyl-acetate fraction of Acacia hydaspica R. Parker
Source: Chem Cent J. 2018 Jan 25;12:5. doi: 10.1186/s13065-018-0373-x (PMC5785459; doi:10.1186/s13065-018-0373-x)
Supplement: Supplementary file 2 — Additional file 2: Figure S2. 1H-NMR spectrum of A. hydaspica compounds. [file 13065_2018_373_MOESM2_ESM.docx]

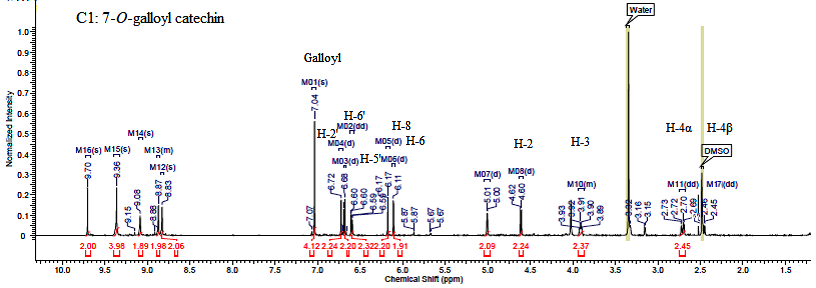


Figure S2: 1H NMR spectrum of GC (C1).Solvent: DMSO-d6, Frequency (MHz):599.67, Nucleus: H, Temperature: 25^o^C, Pulse sequence: s2pul, Acquisition time (sec):1.7039, Number of transits: 16, Original point count: 32768, Spectrum offsets (Hz): 3598.0154, Spectrum type: Standard, sweep width (Hz):9615.38.


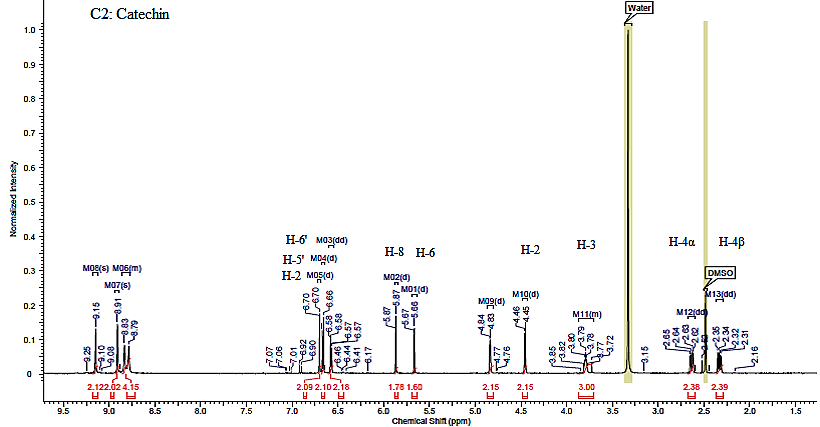


Figure S3: 1H NMR spectrum of C (C2).Solvent: DMSO-d6, Frequency (MHz):599.67, Nucleus: H, Temperature: 25^o^C, Pulse sequence: s2pul, Acquisition time (sec):1.7039, Number of transits: 32, Original point count: 16384, Spectrum offsets (Hz): 3598.0154, Spectrum type: Standard, sweep width (Hz):9615.38


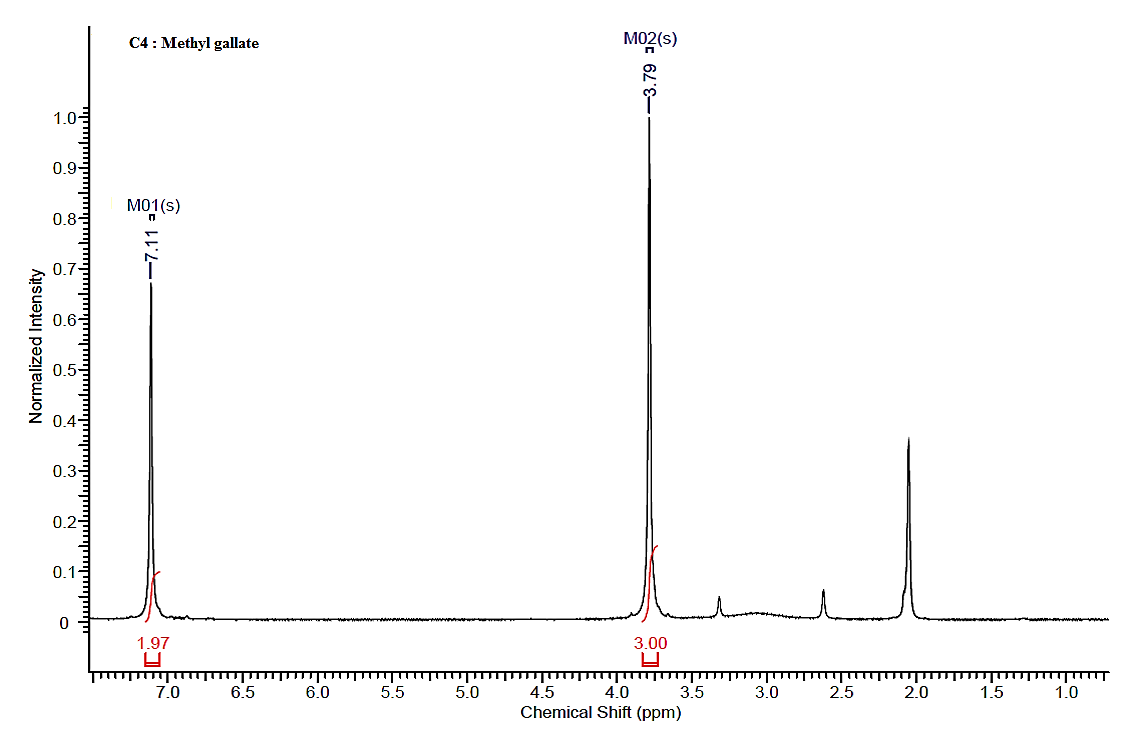


C 3: Methyl gallate

Figure S4: 1H NMR spectrum of MG (C4). Solvent: acetone-d6, Frequency (MHz):599.67, Nucleus: H, Temperature: 25^o^C, Pulse sequence: s2pul, Acquisition time (sec):1.704, Number of transits: 16, Original point count: 16384, Spectrum offsets (Hz): 3598.0154, Spectrum type: Standard, sweep width (Hz):9615.4
